# Elicitation of Induced Resistance against *Pectobacterium carotovorum* and *Pseudomonas syringae* by Specific Individual Compounds Derived from Native Korean Plant Species 

**DOI:** 10.3390/molecules181012877

**Published:** 2013-10-16

**Authors:** Geun Cheol Song, Shi Yong Ryu, Young Sup Kim, Ji Young Lee, Jung Sup Choi, Choong-Min Ryu

**Affiliations:** 1 Molecular Phytobacteriology Laboratory, Superbacteria Research Center, KRIBB, Daejeon 305-806, Korea; E-Mail: song@kribb.re.kr; 2 Biosystems and Bioengineering Program, School of Science, University of Science and Technology, Daejeon 305-333, Korea; 3 Korea Research Institute of Chemical Technology, P.O. Bos 107, 141 Gajeong-ro, Yuseong, Daejeon 305-600, Korea; E-Mails: syryu@krict.re.kr (S.Y.R.); yskim@krict.re.kr (Y.S.K.); jiyoung@krict.re.kr (J.Y.L.); jschoi@krict.re.kr (J.S.C.); 4 Graduate School of New Drug Discovery and Development, Chungnam National University, Daejeon 305-764, Korea

**Keywords:** capsaicin, induced resistance, jaceosidin, plant extracts, systemic acquired resistance

## Abstract

Plants have developed general and specific defense mechanisms for protection against various enemies. Among the general defenses, induced resistance has distinct characteristics, such as broad-spectrum resistance and long-lasting effectiveness. This study evaluated over 500 specific chemical compounds derived from native Korean plant species to determine whether they triggered induced resistance against *Pectobacterium carotovorum* supsp. *carotovorum* (*Pcc*) in tobacco (*Nicotiana tabacum*) and *Pseudomonas syringae* pv. tomato (*Pst*) in *Arabidopsis thaliana*. To select target compound(s) with direct and indirect (volatile) effects, a new Petri-dish-based *in vitro* disease assay system with four compartments was developed. The screening assay showed that capsaicin, fisetin hydrate, jaceosidin, and farnesiferol A reduced the disease severity significantly in tobacco. Of these four compounds, capsaicin and jaceosidin induced resistance against *Pcc* and *Pst*, which depended on both salicylic acid (SA) and jasmonic acid (JA) signaling, using *Arabidopsis* transgenic and mutant lines, including *npr1* and NahG for SA signaling and *jar1* for JA signaling. The upregulation of the *PR2* and *PDF1*.*2* genes after *Pst* challenge with capsaicin pre-treatment indicated that SA and JA signaling were primed. These results demonstrate that capsaicin and jaceosidin can be effective triggers of strong induced resistance against both necrotrophic and biotrophic plant pathogens.

## 1. Introduction

Plants have developed an array of defense mechanisms, including systemic acquired resistance (SAR) and induced systemic resistance (ISR). SAR and ISR comprise plant innate immune responses to a variety of enemies, such as insects and microbial pathogens [1]. Ross first proposed the concept of SAR after identifying the plant characteristics that facilitate self-defense against broad-spectrum pathogens based on challenge experiments using the necrotizing plant virus, *Tobacco mosaic virus*, in tobacco plants that possessed the *N*-gene [2]. The SAR pathway is activated after the formation of a necrotic lesion, either as part of the hypersensitive response (HR) or as a symptom of disease [3]. SAR leads to the development of broad-spectrum and long-lasting effectiveness [3,4]. SAR is elicited by biological compounds, including chitins, ergosterols, glucans, lipopolysaccharides, proteins, peptides, salicylic acid, and sphingolipids. In addition to the biological compounds elicited by induced resistance, other chemicals can also trigger induced resistance and the best example is benzo [1,2,3]thiadiazole-7-carbothioic acid *S*-methyl ester (BTH), which is made by Syngenta. BTH was the first commercialized agrochemical to trigger induced resistance and it is known to be effective against a broad spectrum of diseases and insect pests, including oomycetes in wheat and tobacco (*Nicotiana tabacum*), and whitefly in tomato [5]. Studies of the molecular mechanisms that underlie the mode of action of BTH show that it increases the endogenous salicylic acid (SA) levels by activating SA-dependent signaling and biosynthesis pathways in plants [6]. SAR marker genes have been identified and characterized, and their induction is tightly correlated with the onset of induced resistance in uninoculated (systemic) tissues [7]. SA induces a number of defense-related genes, including pathogenesis-related (PR) genes [8]. In *Arabidopsis*, three induced resistance PR marker genes, *i*.*e*., *PR-1*, *PR-2*, and *PR-5*, have been used most often as SA signaling marker genes [7]. Genes have been characterized that encode typical SAR markers in different plant species, which have been used extensively to evaluate the onset of induced resistance [7,9]. Recent studies have demonstrated that the expression of marker genes depends on the specific elicitor of induced resistance and different pathogens [10,11]. Similarly, ISR is activated by root colonization with plant growth-promoting rhizobacteria (PGPR) [12]. The defense signaling of PGPR-mediated ISR is distinct from SAR because ISR requires jasmonic acid (JA)- and ethylene (ET)-dependent pathways [13]. 

In addition to chemical- and microbe-mediated induced resistance, recent studies have reported environmentally safe and effective methods for the management of plant diseases based on plant extracts. A number of plant species have been reported to contain natural substances that protect plants against various pathogens [14]. For example, the anthraquinones [15] and pure emodin [16] isolated from *Rheum tanguticum* have been shown to possess antifungal properties against phytopathogenic fungi [17]. The *in vitro* growth of bacteria was not affected by treatment with a plant extract of *Hedera helix*, while foliage sprays suppressed disease development significantly after inoculation at different dosages against fire blight caused by *Erwinia amylovora* in field experiments [18]. However, most studies of plant extracts have reported the direct antagonism-based inhibition of pathogen growth. Although plant extracts provide a new means of controlling plant pathogens, a major drawback is the long period required to identify and isolate specific determinants [19]. Thus, alternative approaches are required for screening specific compounds from plant extracts. 

Noutoshi *et al*. screened a commercially available library of 10,000 defined single chemicals, including plant-derived extracts, and selected four sulfonamides and five imprimatins that elicited induced resistance in *Arabidopsis* plants [20]. This study demonstrated that the large-scale screening of chemicals from biological materials, including plant compounds, could be a useful tool for identifying promising triggers of induced resistance. However, this study had some drawbacks. First, only *Arabidopsis* was used as a model system. In reality, however, the resistance induced in *Arabidopsis* is not always replicated in crop plants [21]. For example, a previous study showed that a model legume and *Arabidopsis* exhibited different induced resistance responses to *Rhizoctonia solani* [22,23]. Furthermore, they only assessed a biotrophic pathogen as a model pathosystem, whereas it is well-known that necrotrophic pathogens are also economically important diseases in the field [24]. For example, necrotrophic fungal pathogens, such as *Sclerotinia* and *Botrytis*, cause overall economic losses of >$200 million and €10–100 billion per year in the U.S. and Europe, respectively [25]. In addition, necrotrophic bacterial pathogens cause substantial economic losses. In particular, the soft rot caused by *Pectobacterium* *carotovorum* causes greater losses of overall production than any other bacterial disease [26]. Extensive research has been conducted to understand the plant immune responses that might overcome necrotrophic pathogens, which have shown that biotrophic pathogens are regulated by SA-dependent defense responses, whereas necrotrophic pathogens are regulated by jasmonate- and ethylene-dependent defense responses in plants. Finally, previous studies did not test whether the volatile effects of chemicals elicited induced resistance, although it is well-known that many natural chemicals are effective in their gaseous forms [27,28,29]. Evidence that the volatile substances released by plants can induce responses in other plants has been increasing steadily. For example, volatile compounds, such as methyl jasmonate (MeJA), methyl salicylate (SA), isoprenoids and green leafy volatiles (GLVs), were reported to elicit induced resistance in *Arabidopsis* [30], lima beans [31,32], and tomatoes [33], after the plants experienced mechanical wounding or pathogen/herbivore attacks. 

Based on previous research, the objectives of the present study were: (1) to develop a rapid disease assay system for the simultaneous testing of two application methods, *i*.*e*., direct treatment and volatile emission, using a specific plant-derived compound; and (2) to characterize the SAR induced by specific compound(s) in tobacco and *Arabidopsis* against both necrotrophic and biotrophic pathogens. To achieve the first objective, a Petri-dish-based screening system with four compartments (hereafter referred to as an “X-plate”) was used to screen plant compounds that triggered induced resistance. A plant chemical library was constructed by extracting and identifying single compounds from native Korean plants in the Korea Research Institute of Chemistry. The library contained compounds from 8,271 Korean plant species. The geographical location of Korea means that it has a rich diversity of plants with a high proportion of endemic species. Initially, 528 specific compounds were purified from the extracts of various plant materials and applied to the root systems of tobacco and *Arabidopsis* seedlings. Next, *Pectobacterium carotovorum* subsp. *carotovorum* (*Pcc*) for tobacco and *Arabidopsis* and *P*. *syringae* pv. tomato DC3000 (*Pst*) for *Arabidopsis* were used to challenge the leaves to avoid direct antagonistic interactions between the inoculated plant compounds and pathogen challenge at the same site. The defense signaling pathways affected by these compounds were determined using *npr1* and NahG plants for the SA signaling pathway and *jar1* plants for the JA signaling pathway. Thus, the present study developed a new strategy for screening plant-derived compounds and used chemical libraries to identify specific compounds that induce resistance to biotrophic and necrotrophic pathogens. Furthermore, specific compounds derived from native Korean plant extracts may be useful resources for eliciting induced resistance to various plant pathogens. 

## 2. Results and Discussion

### 2.1. Screening Specific Compounds from Plant Extracts that Elicited Induced Resistance in the X-Plate System

**Figure 1:**
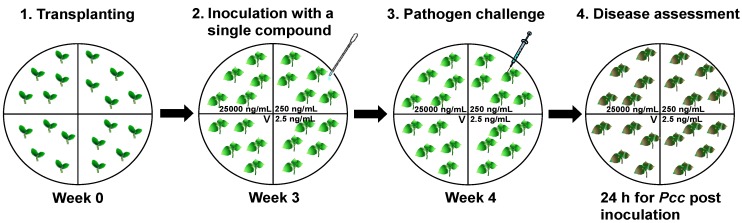
Screening method used to identify specific compounds that elicited induced resistance to *Pectobacterium carotovorum* subsp. *carotovorum* (*Pcc*). Specific compounds were tested using the X-plate system to assess their capacity to induce resistance to *Pcc*. Seven days after direct and indirect (volatile) treatment with the compound solution at three concentrations, *i*.*e*., 25,000, 250, and 2.5 ng/mL, five leaves per plant were drop-inoculated with 20 μL of *Pcc* suspension at 1 × 10^8^ cfu/mL on *Nicotiana tabacum* cv. Xanthi-nc. At 24 h post-inoculation (hpi), the severity of the disease symptoms caused by *Pcc* was assessed. The letter “V” shown on the figure indicates a volatile effect because compounds in the other three compartments volatilized into the air and reached the untreated compartment.

**Figure 2:**
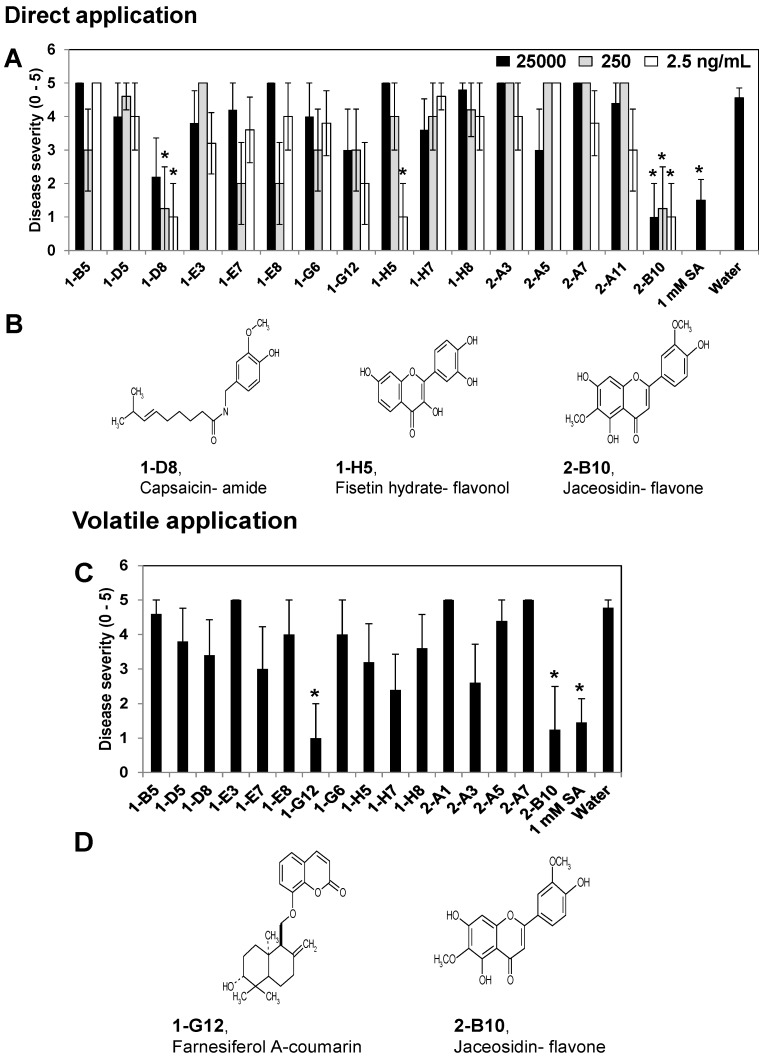
Analysis of the elicitation of induced resistance to *P*. *carotovorum* subsp. *carotovorum* in tobacco by specific compounds using the X-plate system. The symptoms of disease severity (0, no symptoms; 1, mild yellowing of the inoculated leaf; 2, partial softening or collapse of the leaf at the inoculation site; 3, almost complete softening or collapse of the leaf at the inoculation site; 4, intensification of leaf soft rot on other leaves; 5, complete plant collapse) were recorded 24 h after pathogen challenge at 10^8^ cfu/mL. The induced resistance elicited by specific compounds was tested by direct application (**A**) and volatile application (**C**) in *Nicotiana tabacum* cv. Xanthi-nc. The chemical structures of specific screened compounds are shown, *i*.*e*., capsaicin-amide, fisetin hydrate-flavonol, and jaceosidin-flavone (**B**), which were screening by direct application, and farnesiferol A-coumarin and jaceosidin-flavone (**D**), which were screened based on their volatile effects. The asterisks in (**A**) and (**C**) indicate statistically significant differences compared with water-treated control plants (*p* = 0.05). The error bars represent the means ± SEM. The values represent the means of five replicates per treatment, with one seedling per replicate. The experiments were repeated three times and produced similar results.

To evaluate the induction of resistance by specific compounds derived from plant extracts, the present study used two methodological criteria to analyze the dose-dependent response and potential volatilization of each compound, because previous studies of plant extracts [17] and chemical compounds have shown that induced resistance is dose-dependent or elicited by gaseous forms of chemicals [34,35]. For example, volatile organic compounds (VOCs), such as trans-2-hexenal, hexanal, and *cis*-3-hexenal, which are released by wounded plant tissues, have been shown to induce phenylpropanoid-related genes that are known to be involved in plant defense responses [36,37]. The application of green leaf volatiles, *i*.*e*., (*E*)-2-hexenal, (*Z*)-3-hexenal, (*Z*)-3-hexenol, or *allo*-ocimene (2,6-dimethyl-2,4,6-octatriene), reduced disease development when plants were inoculated with *Botrytis cinerea*. Thus, previous studies suggest that volatile treatment can increase the systemic defense response of plants via JA-related plant defense signaling [29]. The present study used the X-plate system (Figure 1), where three of the four compartments contained five seedlings and a specific compound at different concentrations. Three different concentrations of each compound, *i*.*e*., 25,000, 250, and 2.5 ng/mL, were applied to the roots directly. The other compartment contained five plants without the compound and was used to evaluate the volatile effect of the compound. It was hypothesized that the plants in the untreated control compartment might be affected if the compounds from the other three compartments were volatilized into the air and reached the untreated compartment. To assess the induction of plant systemic defenses, a necrotrophic bacterium, *Pectobacterium carotovorum* subsp. *carotovorum*, was drop-inoculated, which caused severe soft rot symptom within 24 h in the control plants. This X-plate system facilitated the high-throughput screening of specific compounds by evaluating the direct dose-dependent effects of compounds and the effects of the volatilized compounds. Initially, 528 specific compounds derived from native plants from South Korea were tested at three concentrations, *i*.*e*., 25,000, 250, and 2.5 ng/mL. The samples were screened based on their capacity to induce resistance to *Pcc* in tobacco plants (Figure 1). The 528 specific compounds were purified from the extracts of various plant materials and their nomenclature was defined before they were deposited in the Korea Chemical Bank, KRICT, Daejeon, Republic of Korea [38]. The first round of screening selected 39 compounds from 528 compounds (data not shown). The second round of screening using 39 compounds yielded 16 candidate compounds and a third round of screening using these 16 compounds produced four compounds (Figure 2A,C).

The compounds capsaicin, fisetin hydrate, and jaceosidin were selected after screening in tobacco based on the reduction of the disease severity in at least one compartment that contained the test compound compared with the water control (Figure 2). The disease severity was reduced significantly in capsaicin-treated plants, *i*.*e*., by 2-, 3.5-, and 4.5-fold in tobacco seedlings treated with 25,000, 250, and 2.5 ng/mL, respectively, while jaceosidin treatment reduced the disease severity by 4.5-, 3.5-, and 4.5-fold with 25,000, 250, and 2.5 ng/mL, respectively, compared with the control treatment. The three different concentrations of jaceosidin reduced the symptoms of soft rot. However, fisetin hydrate only reduced the disease severity with the 2.5 ng/mL treatment. The 1 mM SA treatment (as a positive control) reduced the development of symptoms by 3-fold compared with the control treatment (Figure 2A). Farnesiferol A and jaceosidin were selected because they significantly reduced the development of symptoms based on the disease severity in the tobacco seedlings, while they also had a volatile effect on the untreated compartment (Figure 2C,D). Thus, jaceosidin significantly suppressed the disease symptoms with direct and indirect (volatile) treatments (Figure 2A,C). The subsequent experiments used 250 and 2.5 ng/mL doses of capsaicin and jaceosidin, respectively, because they produced consistent reductions in the disease severity. The chemical structures of capsaicin and jaceosidin are shown in Figure 2B,D.

To the best of our knowledge, this is the first study to report that fisetin hydrate, jaceosidin, and farnesiferol A increase plant systemic defenses (induced resistance). Fisetin hydrate, a naturally occurring flavonoid, which is common in various vegetables and fruits [39], possesses antioxidative [40], anti-inflammatory [41], and antiproliferative effects [42,43]. Jaceosidin, a secondary metabolite in the flavonoid biosynthesis pathway, has menstrual function regulation, anti-aging, and cancer cell inhibition effects [44,45]. Farnesiferol A is one of the sesquiterpene coumarin compounds, which have various biological activities, such as anti-HIV, anti-tumor, anti-hypertension, anti-arrhythmia, anti-inflammatory, anti-osteoporosis, antiseptic, and analgesic effects. In particular, several studies of farnesiferol A have described the antiviral activity of this compound in humans [46]. A recent study showed that capsaicin induced plant defenses against a pathogenic fungus [47]. Capsaicin was applied to plant roots where it had a direct antimicrobial effect, while it also had a chitinase activity and elicited the expression of several defense-related genes, e.g., *CaSC1*, *CaCHI2*, and *CaBGLU*, against *Verticillium dahliae* [47]. Therefore, the four compounds selected in this study, *i*.*e*., capsaicin, fisetin hydrate, farnesiferol A, and jaceosidin, elicited induced resistance to pathogens so further experiments were conducted to determine their effects on the expression of several defense-related genes. 

### 2.2. Elicitation of Induced Resistance by Capsaicin and Jaceosidin in Col-0, *npr1*, NahG, *jar1-1*, and *etr1-3* Plants

**Figure 3:**
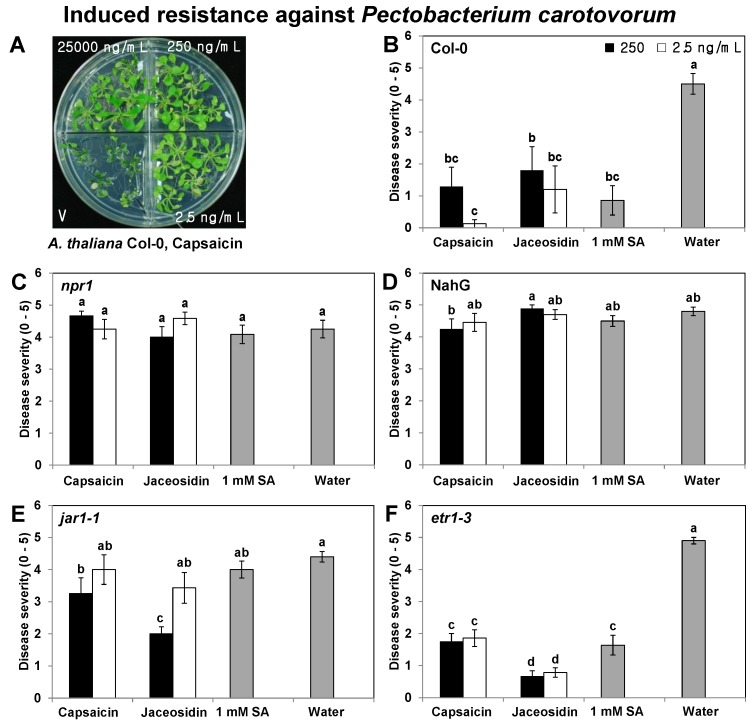
Induced resistance to *P*. *carotovorum* subsp. *carotovorum* in *Arabidopsis* *thaliana* Col-0, the defense signaling-deficient line *npr1*, NahG, *jar1-1*, and *etr1-3* after treatment with capsaicin and jaceosidin. Image showing the infection of *Arabidopsis* seedlings by *P*. *carotovorum* subsp. *carotovorum* at 24 h after pathogen challenge on the leaves of 4-week-old *Arabidopsis*, which had been treated with capsaicin and jaceosidin at 7 days previously (**A**). *Arabidopsis* seedlings Col-0 (**B**), *npr1* (**C**), NahG (**D**), *jar1-1* (**E**), and *etr1-3* (**F**) were treated with 250 and 2.5 ng/mL concentrations of capsaicin and jaceosidin in the X-plate system and compared with control plants inoculated with sterile water. The disease severity symptoms (0, no symptoms; 1, mild yellowing of the inoculated leaf; 2, partial softening or collapse of the leaf at the inoculation site; 3, almost complete softening or collapse of the leaf at the inoculation site; 4, intensification of leaf soft rot on other leaves; and 5, complete plant collapse) were measured 1 day after inoculation of the pathogen at 10^8^ cfu/mL. The values represent the means of five replicates per treatment, with one seedling per replicate. The different letters in panels B to F indicate significant differences among treatments (*p* = 0.05, according to LSD test). The error bars represent the means ± SEM.

The disease protection effects of 250 and 2.5 ng/mL capsaicin and jaceosidin were tested in wild-type Col-0 and three *Arabidopsis* mutants, *i*.*e*., *npr1*, *jar1-1*, and *etr1-3*, as well as a transgenic line of NahG plants, to identify the defense signaling pathways affected by these compounds. In the three mutants and the transgenic line, SA signaling-defective *npr1* and NahG abolished the induced resistance elicited by the 250 and 2.5 ng/mL doses of capsaicin and jaceosidin (Figure 3C,D). Induced resistance was only elicited in the JA-insensitive mutant *jar1-1* plant by 250 ng/mL capsaicin and jaceosidin (Figure 3E), whereas treatment of the wild-type Col-0 and ethylene (ET) receptor mutant *etr1-3* plant with the two concentrations of capsaicin and jaceosidin consistently reduced the disease severity caused by *Pcc* (Figure 3B,F). The disease severity was reduced by 3.5- and 4.6-fold with the capsaicin treatment, and by 2.6- and 3.8-fold with the 250 and 2.5 ng/mL jaceosidin treatments, respectively, in the wild-type *Arabidopsis thaliana* Col-0 plant. The disease severity in 1 mM SA-treated plants (positive control) was reduced by 5-fold compared with the control treatment (Figure 3B). In the *etr1-3* mutant plant, the disease severity was reduced by 2.8- and 2.6-fold with the 250 and 2.5 ng/mL capsaicin treatments, respectively, and by 7.4- and 6.4-fold with the 250 and 2.5 ng/mL jaceosidin treatments, respectively. The disease severity in the 1 mM SA-treated plants (positive control) was reduced by 3.1-fold compared with the control treatment (Figure 3F). These results demonstrate that the SA and JA signaling pathways were activated by 250 ng/mL doses of capsaicin and jaceosidin. 

### 2.3. Elicitation of Induced Resistance in Col-0, NahG, *npr1*, *jar1-1*, and *etr1-3* Plants by Capsaicin and Jaceosidin against the Biotrophic Pathogen *P. syringae* pv. tomato

**Figure 4:**
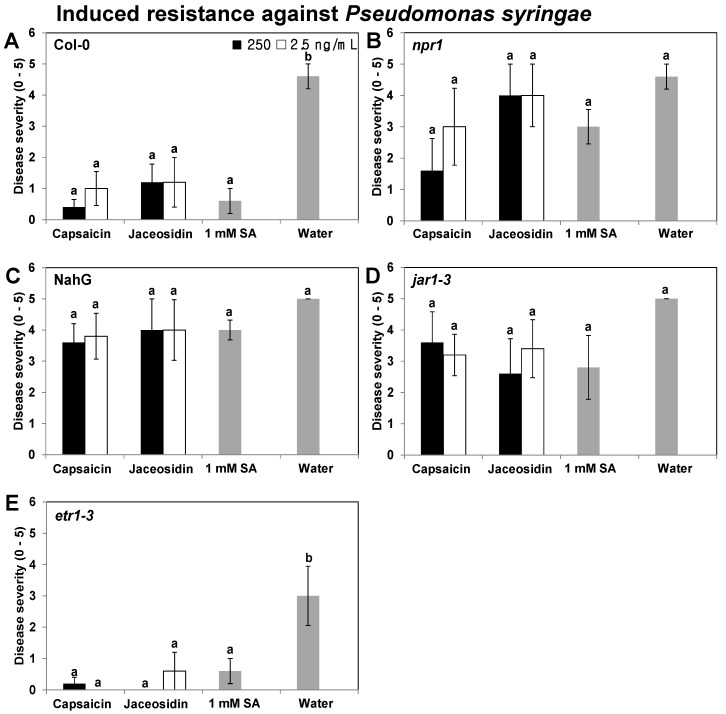
Elicitation of induced resistance in *A.* *thaliana* wild type and mutants against *P*. *syringae* pv. tomato by capsaicin and jaceosidin. Three-week-old *Arabidopsis* Col-0 (**A**), *npr1* (**B**), NahG (**C**), *jar1-1* (**D**), and *etr1-3* (**E**) seedlings were treated with capsaicin and jaceosidin in the X-plate and compared with control plants, which were inoculated using sterile water. Seven days after treatment with the two compounds, all plants were drop-inoculated with *Pst*. The disease severity symptoms (0, no symptoms; 1, mild yellowing of the inoculated leaf; 2, extensive yellowing; 3, brown necrotic lesions; 4, intensification of leaf necrosis; and 5, severe chlorosis) were measured 7 days after inoculation with the pathogen at 10^8^ cfu/mL. The values represent the means of five replicates per treatment, with one seedling per replicate. The different letters in panels A to E indicate significant differences among treatments (*p* = 0.05, according to LSD test). The error bars represent the means ± SEM.

To avoid a shortcoming of the previous experiment conducted by Noutoshi *et al*., the present study tested the induced resistance to biotrophic pathogens and necrotrophs. The elicitation of the plant defense signaling pathway against biotrophic pathogens was tested by eliciting induced resistance using 250 and 2.5 ng/mL of capsaicin and jaceosidin. The induced resistance elicited by capsaicin and jaceosidin (250 and 2.5 ng/mL) was abolished in three mutants and one transgenic line, the SA signaling-related mutant *npr1*, the transgenic line NahG, and the JA-resistant mutant *jar1-1* plants (Figure 4B,E), whereas capsaicin and jaceosidin consistently elicited induced resistance in the wild-type Col-0 and the ET receptor mutant *etr1-3* plants (Figure 4A,E). In the wild-type Col-0, the disease severity was reduced by 11.5- and 4.6-fold with the 250 and 2.5 ng/mL capsaicin treatments, and by 3.8- and 3.8-fold with the 250 and 2.5 ng/mL jaceosidin treatments, respectively. The disease severity in the 1 mM SA-treated plants (positive control) was reduced by 7.7-fold compared with the control treatment (Figure 4A). The disease severity was reduced by 15- and 30-fold with the 250 and 2.5 ng/mL capsaicin treatments in *etr1-3* plants, and by 30- and 5-fold with the 250 and 2.5 ng/mL jaceosidin treatments, respectively. The disease severity in 1 mM SA-treated plants (positive control) was reduced by 5-fold compared with the control treatment in *etr1-3* plants (Figure 4E). These results demonstrate that the SA and JA signaling pathways were activated by 250 and 2.5 ng/mL capsaicin and jaceosidin, which indicates that induced resistance effects against biotrophs and necrotrophs are elicited by capsaicin and jaceosidin, thereby showing that the defenses against both types of pathogens depend on similar defensive signaling pathway. To eliminate the possibility that the translocation of capsaicin and jaceosidin had direct inhibitory effects against *Pcc* and *Pst*, a bioassay was carried out using the paper disc assay method [48]. Drop-inoculation using different concentrations of capsaicin and jaceosidin, *i*.*e*., 25,000, 250, and 2.5 ng/mL, did not inhibit the growth of *Pcc* and *Pst* on the medium (data not shown). These results indicate that the reduction in the *Pcc* and *Pst* symptoms during infection were not a direct consequence of the inhibition of bacterial growth, despite the possible translocation of capsaicin and jaceosidin from the roots to the shoots and leaves.

### 2.4. Expression of Defense-Related Genes in Response to Capsaicin

**Figure 5:**
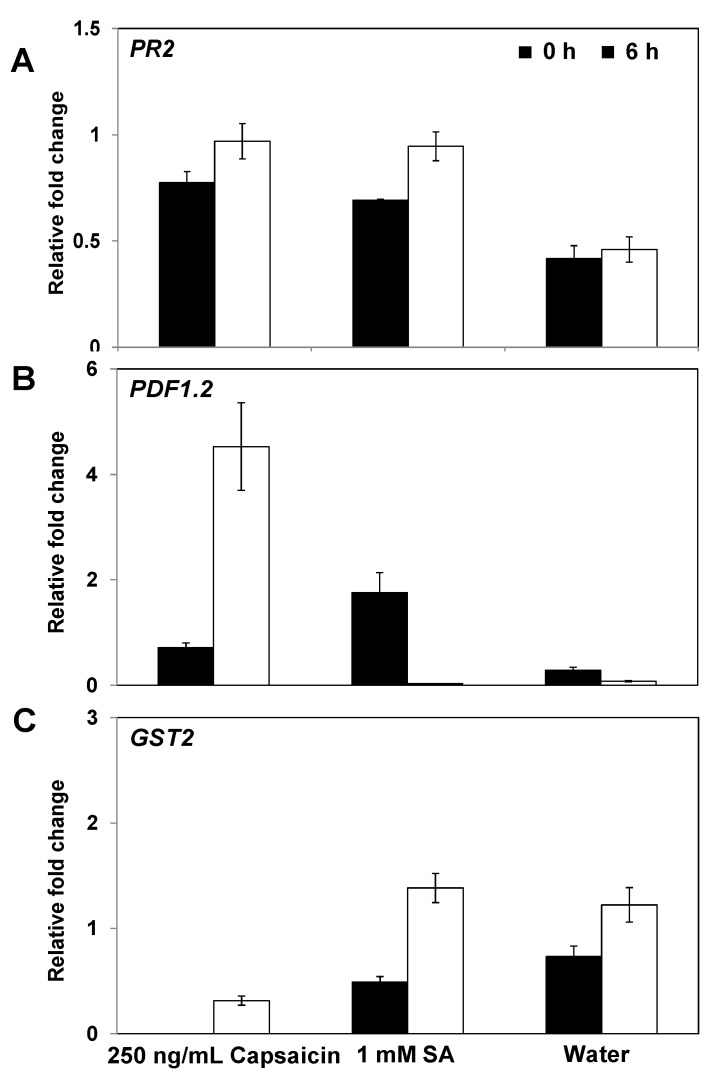
Expression of *PR2*, *PDF1*.*2*, and *GST2* in response to capsaicin treatment. The expression levels of the *Arabidopsis* resistance genes *PR2* (**A**), *PDF1*.*2* (**B**), and *GST2* (**C**)were assessed by quantitative RT-PCR (qRT-PCR) at 0 and 6 h after *Pst* challenge in plants that had been pretreated with 250 ng/mL capsacin. The qRT-PCR results indicated that the induction of *PR2* (SA signaling), *PDF1*.*2* (JA signaling), and *GST2* (ethylene signaling) expression occurred. The error bars represent the means ± SEM and there was a sample size of *N* = 5 plants per treatment. The housekeeping gene *AtActin* was used as a control. The experiment was repeated twice and similar results were obtained.

Quantitative RT-PCR (qRT-PCR) was used to investigate the defense-related genes of *Arabidopsis*, including *PR2* (SA response), *PDF1*.*2* (JA response), and *GST2* (ET response), to determine the mode of action of induced resistance signaling by capsaicin. Capsaicin was selected to evaluate the transcriptional expression of defense-related gene by qRT-PCR to avoid any additional effects due to the volatilization of compounds in the induced resistance assay. Jaceosidin was excluded from further analyses because the compound elicited induced resistance by direct application and via its volatile effects, which suggested that the expression of defense-related genes occurred with both effects. It was found that 250 and 2.5 ng/mL capsaicin failed to elicit induced resistance in *npr1*, NahG, and *jar1* plants, with the exception of the 250 ng/mL treatment of *jar1* (Figure 3C–E). Thus, defense signaling marker genes were analyzed to address this discrepancy. The *PR2* gene, a SA signaling marker gene, was significantly upregulated by as much as 2.1-fold, and the JA signaling marker gene *PDF1*.*2* was significantly upregulated by as much as 64.6-fold, in plants treated with 250 ng/mL capsaicin compared with the water control treatment at 6 hpi. These results strongly suggest that SA and JA signaling are involved in the induced resistance against *Pst* elicited by capsaicin (Figure 5A,B). However, the transcriptional expression of the ET signaling marker gene *GST2* did not differ greatly from the water control at 0 and 6 hpi (Figure 5C). The 1 mM SA treatment increased the expression of *PR2* in *Arabidopsis* tissues but did not affect the *PDF1*.*2* or *GST2* expression levels. Overall, these results indicate that SAsignaling was required for induced resistance activated by only 250 ng/mL but not by 2.5 ng/mL capsaicin (Figure 3E). Treatment with 250 ng/mL strongly upregulated the SA marker gene, *PR2*, which indicates that SA signaling was induced, even in the *jar1* mutant (Figure 5).

Interestingly, the SA response marker gene *PR2* and the JA response marker gene *PDF1*.*2* were significantly upregulated by capsaicin compared with the water control (Figure 5). However, the induction of the ET/JA-dependent plant defense signaling pathway may be more effective against a necrotrophic pathogen such as *Pcc*, rather than *Pst*, which requires a SA-dependent resistance response [13]. Induced resistance to biotrophic and necrotrophic pathogens was also detected in the wild-type and *etr1-3* line after treatment with 250 and 2.5 ng/mL capsaicin and jaceosidin (Figure 3 and Figure 4). It is not clear how capsaicin and jaceosidin elicited induced resistance against biotrophic and necrotrophic pathogens simultaneously. However, a recent study showed that the exposure of *Arabidopsis* to hexadecane derived from *Paenibacillus polymyxa* E681, a long chain VOC, conferred induced resistance to both *Pcc* and *Pst*, although this study did not explain why hexadecane was essential for the elicitation of a SAR against two types of pathogens. It is possible that interactions between the JA and SA pathways may play important roles in fine-tuning the defense responses [49,50]. Studies of JA/SA cross-talk have discovered important dose-dependent effects on the interactions between the two hormones. For example, JA-induced *PDF1*.*2* expression can be enhanced in *Arabidopsis* by the apoplastic injection of SA at concentrations of up to 350 μM, whereas *PDF1*.*2* expression was reduced at higher SA concentrations [51]. Similarly, the accumulation of *PR1* transcripts in response to 10 μM SA was increased by the application of JA up to 125 μM whereas JA concentrations above 125 μM reduced *PR1* expression [51]. These results indicate that seemingly contradictory JA and SA responses may be indicators of the concentration-dependent interactions of the two signaling molecule pathways. The present study showed that capsaicin induced the expression of SA and JA response marker genes (Figure 5), which demonstrated that it elicited resistance to biotrophic and necrotrophic pathogens (Figure 3 and Figure 4).

Von Rad *et al*. investigated defense-related gene expression in *Arabidopsis* plants treated with several commercially available elicitors, including ‘Neudo Vital’, ‘Bio-S’, and ‘PRORADIX’, which are plant or bacterial extracts. All three plant activators induced SA-dependent and JA-dependent genes [52]. Further evidence also suggests that the SA and JA defense signaling pathways function together [53]. Recently, the disease responses of a *Arabidopsis* *dde2/ein2/pad4/sid2* quadruple mutant, in which all of these pathways were blocked, were analyzed to determine the contributions of JA, ET, and SA during plant infections caused by *Alternaria brassicicola* [54]. The immunity was severely compromised in the quadruple mutant compared with all of the single mutants. Thus, the JA, ET, and SA signaling components make positive contributions to the immunity against *A*. *brassicicola* via complex interactions, which is a conclusion that could not be reached based on the single-mutant [55,56]. Similarly, the results of the present study showed that the induction of both SA and JA signaling were involved in induced resistance to biotrophic and necrotrophic pathogens. It is hoped that further greenhouse and field experiments will broaden our knowledge of the plant protective effects against various pathogens elicited by the specific compounds indentified in the present study.

Induced resistance appears to be another phenomenon that involves interactions between pathogens and plants, where induction occurs when the plant defenses are triggered by a primary infection, although the resistance pathways are only expressed during the actual challenge. 

In contrast to the lack of direct *Pcc* and *Pst* inhibition in the present study, extensive research has demonstrated the antimicrobial and antifungal activities of capsaicin. The antimicrobial effects of untreated and heated extracts from *Capsicum* species (*Solanaceae*) were tested in 15 bacterial species and one yeast species, which showed that the extract produced variable degrees of inhibition in five bacterial species [57]. Thus, some of the beneficial characteristics of capsaicin are related to its antimicrobial and antifungal activities [58,59,60,61]. For the first time, a recent study reported the elicitation of induced resistance by capsaicin. *In planta* tests, where capsaicinoids (capsaicin and *N*-vanillylnonanamide) were applied to the roots, demonstrated that these compounds conferred protection against the pathogenic fungus *Verticillium dahliae*, as well as inducing chitinase activity and the expression of several defense-related genes, such as *CaSC1* (sesquiterpene cyclase), *CaCHI2* (chitinase), and *CaBGLU* (β-1,3-glucanase). In the pepper, capsaicinoids trigger the expression of the sesquiterpene cyclase/5-epi-aristolochene gene, *CaSC1*, which is involved in the synthesis of the phytoalexin capsidiol. Two PR genes were shown to be slightly upregulated by capsaicinoids, *i*.*e*., *CaBGLU* and *ACHI2*, as well as the chitinase activity, before pathogen challenge [47]. Both of these genes encode hydrolytic enzymes, *i*.*e*., a basic β-1,3-glucanase and a basic class II chitinase, respectively. Chitinases and β-1,3-glucanases are biochemical markers of induced resistance, which are involved in the degradation of the cell walls of pathogens [62]. 

Various compounds have been shown to induce resistance and reduce the level of infection caused by subsequent pathogen challenge. It is important to know whether a small or large number of compounds can induce resistance, because this will indicate how many pathways can be activated. Overall, the present study suggests that specific compounds derived from eco-friendly materials can trigger induced resistance and it may be possible to use them to protect crop plants from pathogens in the field.

## 3. Experimental

### 3.1. Plant Preparation and Disease Assay

The seeds of *Nicotiana tabacum* cv. Xanthi-nc and all *Arabidopsis thaliana* plants (Col-0, e*tr1-3*, *jar1-1*, *npr1*, and NahG) were surface-sterilized with 3% sodium hypochlorite, washed four times with sterile distilled water, and maintained at 26 °C and 23 °C, respectively, with a 14 h:10 h light:dark cycle for 3 days until germination in half-strength Murashige and Skoog salt (MS) medium (GIBCO/BRL) containing 0.6% agar and 1.5% sucrose, where the pH was adjusted to 5.8. The tobacco and *Arabidopsis* seedlings were transferred to X-plates. Petri dishes (100 × 15 mm) that contained four compartments (X-plates; Falcon) were prepared with 1/2 MS solid medium, and five germinated *A*. *thaliana* seedlings (2 days old) were transferred to each compartment in the X-plates. Two weeks after transplantation, the seedling roots were treated with 50 μL water as the negative control, 1 mM SA as the positive control, and using different specific compounds derived from plant extracts. The fresh solutions of 528 compounds derived from plant extracts were prepared at three different dilutions, *i*.*e*., 25,000, 250, and 2.5 ng/mL. Seven days after treatment with the specific compounds derived from plant extracts, five leaves per plant were treated with 20 μL drops that contained a suspension of 1 × 10^8^ cfu/mL *Pectobacterium carotovorum* supsp. *carotovorum* SCC1 (*Pcc*) and *Pseudomonas syringae* pv. tomato DC3000 (*Pst*). The severity of the disease symptoms caused by *Pcc* was assessed at 24 h by scoring according to a published method [63]. The severity of the disease symptoms caused by *Pst* were assessed between 5 and 7 days by scoring (0–5) according to a published method [64]. This experiment had a completely randomized design with five replicates and one plant per replicate. The experiment was repeated three times.

### 3.2. Plant Extraction and Identification

The specific compounds isolated from plant extracts were identified as described previously [65]. Briefly, each tissue was harvested and prepared following drying at room temperature for five days under shade conditions. The dried tissues were macerated and extracted with 99.8% methanol at 50 °C. The plant extracts were dried and then aliquoted at 20–50 mg in dimethyl sulfoxide (DMSO) solution for storage at 4 °C until required. The diverse fractionation processes were subjected to silica gel column chromatography and eluted with a mixture of individual solvents to yield 120 mg of each compound as crystals. The spectral data obtained and identified from each specific compound (purity > 99%), *i*.*e*., UV, MS, ^1^H-NMR, and ^13^C-NMR, agreed well with the results of Bhattacharyya and Carvalho [66]. All the dried plant samples were dissolved in 99.99% methanol for dilution. For the screening from 528 compounds, four compounds, capsaicin, fisetin hydrate, jaceosidin, and farnesiferol A with three concentrations, 25,000, 250, and 2.5 ng/mL were assessed the capacity of the induced resistance. The selected two compounds, capsaicin and jaceosidin (250 ng/mL and 2.5 ng/mL) were used for further experiments. The final concentration of methanol was 1% for 250 ng/mL and 0.01% for 2.5 ng/mL of the capsaicin and jaceosidin. 

### 3.3. Direct Inhibition Assay

To test whether capsaicin and jaceosidin had direct inhibitory effects on *Pcc and Pst*, a bioassay was performed using the paper disc assay method [48] where 100 μL of 10^8^ cfu/mL *Pcc* and *Pst* suspension was spread onto Luria-Bertani medium and King’s B medium. Fifty microliters of capsaicin, jaceosidin, and 1 mM SA were pipetted onto the paper discs. The dried paper discs were transferred aseptically onto the surface of the growth medium. Two days later, the discs were checked to determine the development of zones of inhibition. At least three replicate plates were prepared for the assay.

### 3.4. Induced Resistance in *npr1*, NahG, *jar1-1*, and *etr1-3* Plants Using Specific Compounds

To test whether specific compounds derived from the plant extract solution elicited induced resistance via three representative defense signaling pathways, *i*.*e*., JA, ET, and SA, the protective effects against *Pcc* and *Pst* were assessed in wild-type Col-0 and its mutants (including *npr1* and NahG) to assess SA signaling, *jar1-1* to assess JA signaling, and *etr1-3* to assess ET signaling. This experiment had a completely randomized design with five replicates and one plant per replicate. The experiment was repeated three times. The induction of defense genes was investigated by qRT-PCR analysis using the plant leaf tissues from Col-0 at 0 h and 6 hpi.

### 3.5. Quantitative RT-PCR

RT-PCR was performed using a Bio-Rad (Hercules, CA, USA) CFX96 system. The total RNA was isolated from *Arabidopsis* leaf tissues using TRI reagent (Molecular Research Inc., Cincinnati, OH, USA), according to the manufacturer’s instructions. The first-strand cDNA synthesis was conducted using 2 μg of DNase-treated total RNA, oligo-dT primers, and Moloney murine leukemia virus reverse transcriptase (MMLV-RT, Enzynomics, Daejeon, Korea). The PCR reactions were performed according to the manufacturer’s instructions. The expression levels of candidate *Arabidopsis* defense genes were analyzed using the following primers: 5'-CCACTGACACCACGGATAC-3' (*PR2*-F), 5'-AAGGGTAGAGATTCACGAGCAAGG-3' (*PR2*-R), 5'-AATGAGCTCTCATGGCTAAGTTTGCTTCC-3' (*PDF1*.*2*-F), 5'-AATCCATGGAATACACACGATTTAGCACC-3' (*PDF1*.*2*-R), 5'-GCCATTGTTGCAAAAGCAGA-3' (*GST2*-F), and 5'-GCCAAAGACCACTGCCAC-3' (*GST2*-R). The candidate genes were amplified from 100 ng of cDNA by PCR using an annealing temperature of 55 °C. The expression of *Actin* was analyzed using the primers 5'-GTTAGCAACTGGGATGATATGG-3' and 5'-CAGCACCAATCGTGATGACTTGCCC-3' as a control to ensure that equal amounts of RNA were analyzed in each experiment [49,67]. A Chromo4 real-time PCR system (Bio-Rad) was used to perform the qRT-PCR analyses. The reaction mixtures contained cDNA, iQTM SYBR^®^ Green Supermix (Bio-Rad), and 10 pM of each primer. The thermocycler parameters were as follows: initial polymerase activation for 10 min at 95 °C, followed by 40 cycles of 30 s at 95 °C, 60 s at 55 °C, and 30 s at 72 °C. The conditions were determined by comparing the threshold values using a series of dilutions of the RT product, followed by a non-RT template control and a non-template control for each primer pair. Relative RNA quantification was performed with the 2-ΔΔCT method based on the standard errors of the mean values of replicates using Bio-Rad Manager version 2.1 (Bio-Rad CFX Connect). Student’s *t*-test was performed to determine statistically significant differences between treated and untreated samples. The target genes were considered to be differentially expressed at *p* < 0.05. The relative RNA levels were calibrated and normalized against the expression levels of *Actin* mRNA.

### 3.6. Statistical Analysis

The experimental datasets were tested by analysis of variance using JMP version 5.0 (SAS Institute Inc., Cary, NC, USA). The significant effects of treatments were determined based on the magnitude of the F value (*p* = 0.05). When a significant F test was obtained, the means were separated using Fisher’s protected LSD at *p* = 0.05.

## 4. Conclusions

In this study, four specific plant compounds that elicited induced resistance in plants were identified by an extensive screening of native Korea plant extracts, which avoided the problem of long-term fractionation. Our new disease assay system facilitated the rapid identification of promising compounds that elicited induced resistance in tobacco and *Arabidopsis*. The plant-derived compounds capsaicin and jaceosidin elicited strong induced resistance to biotrophic and necrotrophic pathogens. The involvement of specific signaling pathways was confirmed using mutants and transgenic plants, as well as defense marker gene expression analyses. It is concluded that these specific individual plant compounds are promising natural resources for protecting plants against pathogens by enhancing plant defenses. 
